# Editorial: An update on neurological disorders post COVID-19 infection vol 2: cardiovascular effects, neuro-cardiac and neuro-respiratory autonomic dysfunctions

**DOI:** 10.3389/fneur.2025.1611381

**Published:** 2025-05-30

**Authors:** Beatrice Paradiso, Giulia Ottaviani, Clara Limback, Dainius Haroldas Pauza, Gaetano Thiene

**Affiliations:** ^1^Department of Biomedical, Surgical and Dental Sciences, Faculty of Medicine and Surgery, Lino Rossi, Research Center, University of Milan, Milan, Italy; ^2^Anatomic Pathology Unit, Angel Hospital Venice, Venice, Italy; ^3^Oxford University Hospitals, NHS Trust, Oxford, United Kingdom; ^4^Institute of Anatomy, Lithuanian University of Health Sciences, Kaunas, Lithuania; ^5^Cardiovascular Pathology, Department of Cardiac, Thoracic, Vascular Sciences and Public Health, University of Padua, Padua, Italy

**Keywords:** COVID 19, cardiovascular diseases, autonomic dysfunction, mental diseases, respiratory diseases (RESPD)

Coronavirus disease 2019 (COVID-19), caused by SARS-CoV-2, has resulted in a variety of long-term problems defined as post-acute sequelae of SARS-CoV-2 infection (PASC). These consequences can include neurological, cardiovascular, autonomic, and immunological dysfunctions, with symptoms ranging from fatigue and cognitive impairment to dysautonomia and immune-mediated vascular damage. Common clinical symptoms include “brain fog,” exercise intolerance, and post-exertional malaise. Similarities to diseases such as myalgic encephalomyelitis/chronic fatigue syndrome (ME/CFS) have also been hypothesized. The underlying pathophysiology is assumed to be a complex interaction of central and autonomic nervous system dysfunction, chronic inflammation, immunological dysregulation, and vascular impairment.

This Research Topic brings together a wide range of studies that illuminate the intricate mechanisms behind PASC and reflect the growing scientific endeavor to understand its systemic impact. One of the most fascinating areas of investigation is the impact of SARS-CoV-2 on the neurological system, particularly in terms of cognitive and emotional health. Talkington et al. presented a complete analysis of neuroimaging findings in long COVID patients, focusing on neuroinflammation, vascular impairment, and blood-brain barrier disruption. These pathophysiological aspects may underlie the cognitive impairments commonly reported by patients, emphasizing the necessity for coordinated diagnostic techniques.

Cahan et al. expanded on this picture by investigating how fatigue and mood problems interact with cognitive deficiencies, emphasizing the significance of treating both the neurological and psychosocial aspects of long COVID. Their contribution emphasizes the complexities of the clinical picture and the importance of multidisciplinary treatment. Pommy et al. extended their investigation by investigating changes in cerebrovascular reactivity in the elderly, employing modern neuroimaging methods to uncover significant changes across functional brain networks. Their findings provide new insights into how vascular dysfunction may manifest in cognitive symptoms, particularly in aging populations, and suggest that cerebrovascular dysregulation may serve as both a marker and a cause of cognitive loss in PASC.

Another well-documented effect of long COVID is impairment of the autonomic nervous system. Several studies have focused on this topic, shedding light on its clinical symptoms and therapeutic applications. Pierson et al. provided a comprehensive overview of pharmaceutical alternatives for postural orthostatic tachycardia syndrome (POTS), a common finding in PASC patients, emphasizing the potential of beta-blockers, ivabradine, and midodrine. Cantrell et al. presented a distinct post-COVID POTS phenotype characterized by concomitant migraine, fatigue, and gastrointestinal problems, highlighting the frequent overlap of autonomic and systemic symptoms. Liviero et al. provided crucial longitudinal data suggesting that even those patients with mild illness may undergo sustained changes in autonomic regulation, as demonstrated by changes in heart rate variability. These findings challenge previous assumptions that only severe COVID-19 cases are at long-term risk and call for vigilance in post-infection follow-up.

In their report of an immune-mediated example of orthostatic hypotension, Theiler et al. emphasized the importance of identifying underlying pathophysiological factors in dysautonomia patients. Their findings show that immunological mechanisms may play a greater role in post-COVID autonomic problems than previously thought, necessitating additional research into autoantibody patterns and inflammatory mediators.

**Figure 1:**
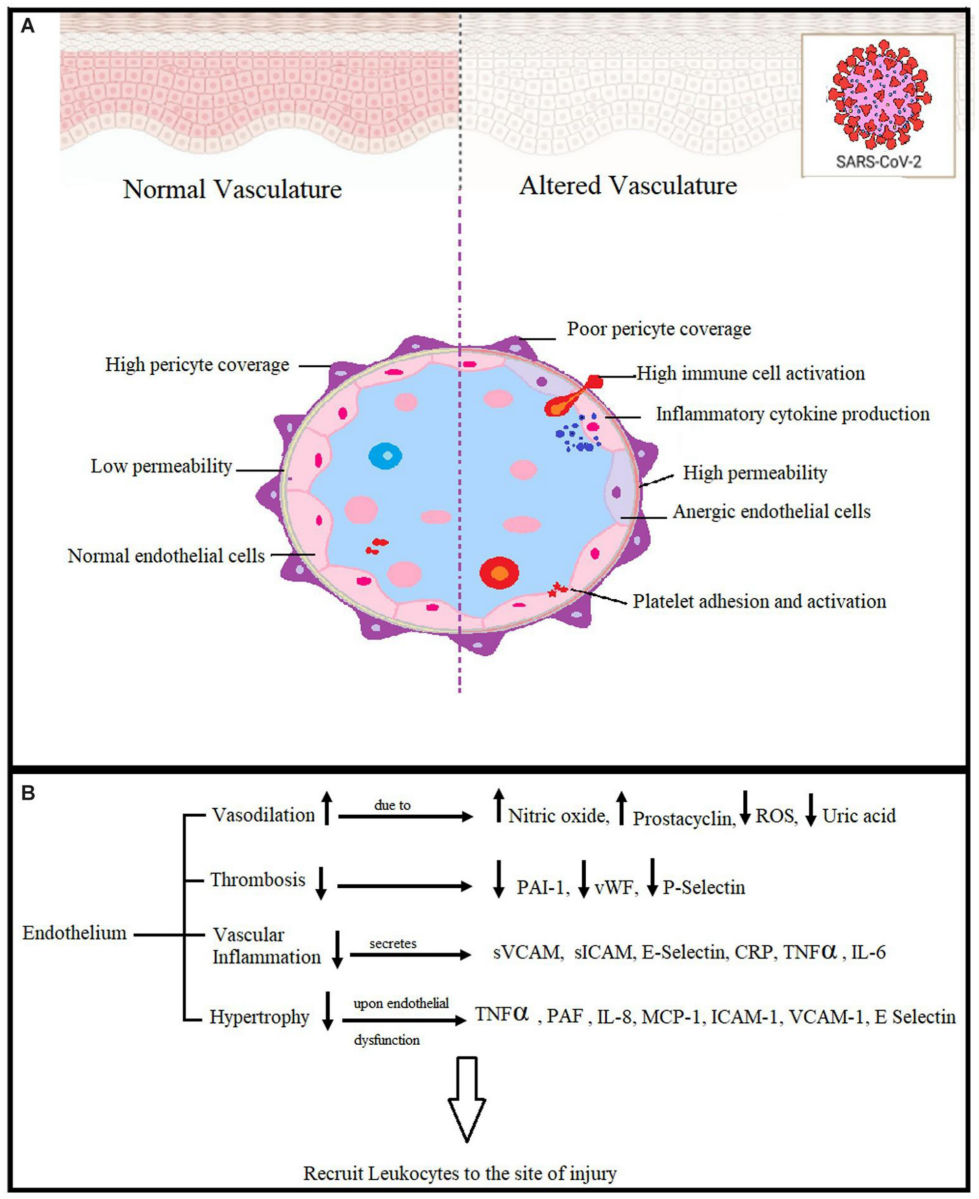
**(A,B)** Adapted from Mehboob, von Kries et al., with permission.

The Research Topic of immunological and vascular interactions is critical to understanding extended COVID. Mehboob, Oehme et al. and Mehboob, von Kries et al. conducted two COMPLEMENTARY studies on the role of Substance P and ACE-II dysregulation in prolonging endothelial damage and inflammation. Their research helps to explain how neuropeptide signaling and poor vascular homeostasis can contribute to persistent symptoms. An especially informative graph from their analysis (Figure 1) depicts the chain of events leading to endothelial injury, hypoxia, and neuroinflammation, potentially providing a unifying mechanism for cognitive symptoms in neuro-PASC. The findings of Pommy et al. support this vascular hypothesis by emphasizing the importance of a diminished cerebrovascular response as a source of cognitive disruption and identifying potential interventional targets for future treatment trials.

Systemic inflammation and metabolic imbalance have also been identified as important contributors to PASC. Rus explored the interaction of the serotonin and kynurenine pathways, hypothesizing that dysfunction in these metabolic circuits may be responsible for many of the neuropsychiatric symptoms seen in long COVID. The kynurenine pathway is known to be implicated in neuroinflammation and neurotoxicity, and its dysregulation may operate as a link between immune activation and mental health issues. This line of research opens the door to new biomarkers and tailored treatments to restore metabolic and immunological balance.

Clinical outcomes remain a major source of concern, particularly for disadvantaged groups. Desouky et al. analyzed hospitalized patients with pre-existing neurological conditions and found that those with dementia, epilepsy, and chronic headaches had higher mortality rates. These findings highlight the need for better surveillance and targeted care techniques for at-risk individuals during and after COVID-19. The study demonstrates how pre-existing brain vulnerability may increase the risk of systemic infections, emphasizing the importance of integrative care measures.

While understanding the underlying mechanisms of PASC is critical, the importance of encouraging recovery and resilience cannot be overstated. Several articles emphasize the necessity for a comprehensive, personalized approach to post-COVID care. Behavioral and rehabilitative treatments, along with attention to lifestyle and psychological factors, can play an important role in restoring function and quality of life. Understanding why some people heal more fully than others may help to guide future clinical management and research objectives. Longitudinal research and systematic rehabilitation programmes will be required in the coming years to determine the best strategies for addressing these chronic symptoms.

The articles in this Research Topic demonstrate the multidimensional character of PASC and the crucial need for multidisciplinary research and care. These contributions go beyond mere description and chart a course for mechanistic clarity, improved diagnosis, and more effective therapies. They reflect the scientific community's collaborative endeavors not only to study the effects of COVID-19, but also to provide practical tools and pathways for recovery. As editors, we are grateful to the authors and reviewers for their careful work in making this publication possible. We hope that it will educate, inspire, and assist the many professionals and patients who navigate the challenges of post-COVID disorders.

